# Associations of VEGF-D levels with clinical manifestations in lymphangioleiomyomatosis: a cross-sectional analysis of 631 cases

**DOI:** 10.1186/s13023-025-03802-4

**Published:** 2025-05-26

**Authors:** Luning Yang, Hanghang Wang, Chongsheng Cheng, Miaoyan Zhang, Danjing Hu, Yani Wang, Tengyue Zhang, Xiaoxin Zhang, Song Liu, Wenshuai Xu, Junya Liu, Jinrong Dai, Shuzhen Meng, Yanli Yang, Shao-Ting Wang, Xinlun Tian, Kai-Feng Xu

**Affiliations:** 1 Department of Pulmonary and Critical Care Medicine, State Key Laboratory of Complex Severe and Rare Diseases, Peking Union Medical College Hospital, Chinese Academy of Medical Sciences & Peking Union Medical College, Beijing, China; 2 Center for Bioinformatics, National Infrastructures for Translational Medicine, Institute of Clinical Medicine, Chinese Academy of Medical Sciences & Peking Union Medical College, Beijing, China; 3 Department of Pulmonary and Critical Care Medicine, Tsinghua University Affiliated Beijing Tsinghua Changgung Hospital, Beijing, China

**Keywords:** Lymphangioleiomyomatosis, Tuberous sclerosis complex, Vascular endothelial growth factor-D

## Abstract

**Background:**

Lymphangioleiomyomatosis (LAM) is a rare neoplastic disorder characterized predominantly by dyspnea, recurrent pneumothorax, chylous effusion and diffuse pulmonary cystic changes in women. Vascular endothelial growth factor-D (VEGF-D) is an important biomarker for LAM.

**Results:**

This study cohort comprised 631 LAM patients and investigated the correlations between serum VEGF-D levels and clinical manifestations of LAM. The median serum level of VEGF-D was 1452pg/ml (820.0-2659pg/ml) among the study population. Patients with highly-elevated VEGF-D levels exhibited younger age, lower BMI, and a higher prevalence of tuberous sclerosis complex (TSC). Elevated VEGF-D levels were associated with a lower prevalence of pneumothorax and angiomyolipomas (AMLs), and a higher risk for retroperitoneal lymphangioleiomyomas and chylous effusion. Elevated VEGF-D levels were associated with increased High-Resolution Computed Tomography (HRCT) LAM grading, reduced forced expiratory volume in one second (FEV_1_) and diffusion capacity for carbon monoxide (DLco), and decreased arterial oxygen partial pressure. Multiple logistic regression analysis identified age, TSC, AMLs, retroperitoneal lymphangioleiomyomas, chylous effusion, and HRCT grade as independent risk factors for elevated VEGF-D levels. As a diagnostic biomarker for LAM, adding VEGF-D in the diagnostic algorithm enabled the diagnosis of LAM without lung biopsy in 83.5% patients with LAM.

**Conclusions:**

The findings highlighted the pivotal role of serum VEGF-D in LAM pathophysiology and underscore that age, TSC, retroperitoneal LAM, chylous effusion, AMLs, and high HRCT grade were independent risk factors for increased VEGF-D levels. VEGF-D is a valuable biomarker in evaluation of LAM and improve the efficiency of diagnostic algorithm.

**Supplementary Information:**

The online version contains supplementary material available at 10.1186/s13023-025-03802-4.

## Introduction

Lymphangioleiomyomatosis (LAM) is a rare neoplastic disease with dyspnea, recurrent pneumothorax, and diffuse pulmonary cystic changes as the main manifestations primarily in women [1, 2]. The imaging features are pulmonary diffuse cystic changes. The disease can be sporadic (S-LAM) or with the autosomal dominant disorder tuberous sclerosis complex (TSC-LAM). The activation of the mammalian target of rapamycin (mTOR) is central to the pathogenesis of LAM. The mTOR inhibitors, such as rapamycin (sirolimus), can stabilize patients’ lung function and improve their health-related quality of life [3–5].

The clinical guidelines for the diagnosis and management of LAM issued by the American Thoracic Society (ATS) and the Japanese Respiratory Society (JRS) recommended that patients with characteristic chest HRCT changes and the abnormal elevation of serum vascular endothelial growth factor-D (VEGF-D) levels can be diagnosed without lung biopsy [6]. VEGF-D is currently the only biomarker recommended in guidelines for non-invasive serological diagnosis of LAM [6]. VEGF-D is a secreted dimeric glycoprotein expressed in the lung, heart, gut, and skeletal muscle. The binding of VEGF-D to VEGF receptors activates downstream kinases that promote lymphangiogenesis and angiogenesis [7]. Immunohistochemical (IHC) studies have identified that the LAM-associated lymphatic endothelial cells (LECs) express high level of VEGFR-3 (the receptor of VEGF-D). These LECs exhibit enhanced proliferation and migration ability, potentially contributing to the lymphangiogenesis in LAM lesions [8, 9]. The significant clinical values of VEGF-D in diagnosis of LAM and prediction of disease progression and survival have been reported [10–14]. In this work, we wish to re-evaluate the real world VEGF-D data in a cohort with a large sample size of patients, focusing on the interaction of VEGF-D levels and clinical features and how VEGF-D assisted in diagnostic algorithm.

## Methods

### Patient population

This is a single-center, cross-sectional study. We enrolled patients with definite LAM diagnosis registered in a prospective LAM-CHINA cohort (Registration number: NCT03193892, IRB approval issued in 2017) at the Peking Union Medical College Hospital (PUMCH) from April 2017 to November 2023. The diagnosis was based on guidelines by the ATS/JRS [5]. Patients with missing VEGF-D on baseline evaluation were excluded. Clinical data of the patients were collected, and VEGF-D levels were measured simultaneously at the time of enrolment. The LAM-CHINA registry research was approved by the ethical committee of the PUMCH (JS1323). All patients signed the informed consent.

### Clinical evaluation

The following data were gathered: age, time from disease onset to diagnosis, past medical history of tuberculosis or malignancy, body mass index, history of TSC, menopause status, history of cigarette smoking, oxygen therapy, and sirolimus treatment in the past one year. Pulmonary function tests, arterial blood gas at room air, six-minute walking distance (6MWD), and St. George’s Respiratory Questionnaire (SGRQ) [15] were evaluated. Asthma was defined as FEV_1_/FVC <70% and positive for bronchodilation test. The prevalence of retroperitoneal lymphangioleiomyomas, chylous effusion (chylothorax or chyloperitoneum), and the presence of renal angiomyolipomas were determined by radiological assessment or pathology. HRCT of the lungs was performed. The severity of LAM was categorized based on the extent of cystic involvement determined by a pulmonologist with experience. Grade 1 indicated less than one-third of the lung, while Grade 2 between one-third and two-thirds, and Grade 3 more than two-thirds [16]. Chronic oxygen supplementation was defined as oxygen therapy for more than 8 h per day and more than 14 days in total. Pulmonary function tests were conducted according to the guidelines established by the ATS and the European Respiratory Society’s Task Force for the Standardization of Lung Function Testing [17]. The six-minute walking tests were evaluated by the ATS Guidelines [18]. All radiological examinations were interpreted collaboratively by radiologists and respiratory physicians.

### Serum VEGF-D measurement

Serum VEGF-D levels were measured by the enzyme-linked immunosorbent assay kit (R&D Systems, Minneapolis, MN, USA, #DVED00) with an available range of 125–4000 pg/ml. The samples were diluted and retested if they were over 4000pg/ml. VEGF-D was measured simultaneously as the clinical evaluation was performed.

### Statistical analysis

Continuous variables were presented as mean ± standard deviation (SD) or median with interquartile range (IQR, 25th -75th percentiles), while categorical variables were reported as numbers with percentiles. Ordinary one-way analysis of variance (ANOVA) was used to compare continuous variables with normal distribution, and Kruskal-Wallis tests for non-normal distribution. Chi-square tests for trends were used to assess differences in categorical variables. Simple logistic regression was utilized to measure the association between VEGF-D levels and clinical parameters. Pearson correlation coefficients were measured to evaluate the correlation among different clinical parameters. Multiple logistic regression was performed to explore the factors related to VEGF-D. Missing data were imputed using the missForest algorithm in Python [19]. A two-tailed *P* value less than 0.05 was considered statistically significant. Data were analysed using Prism version 9.0.0 (86), October 24, 2020 (1994–2020 GraphPad Software, LLC).

## Results

### VEGF-D levels and their correlation with clinical parameters

**Fig. 1:**
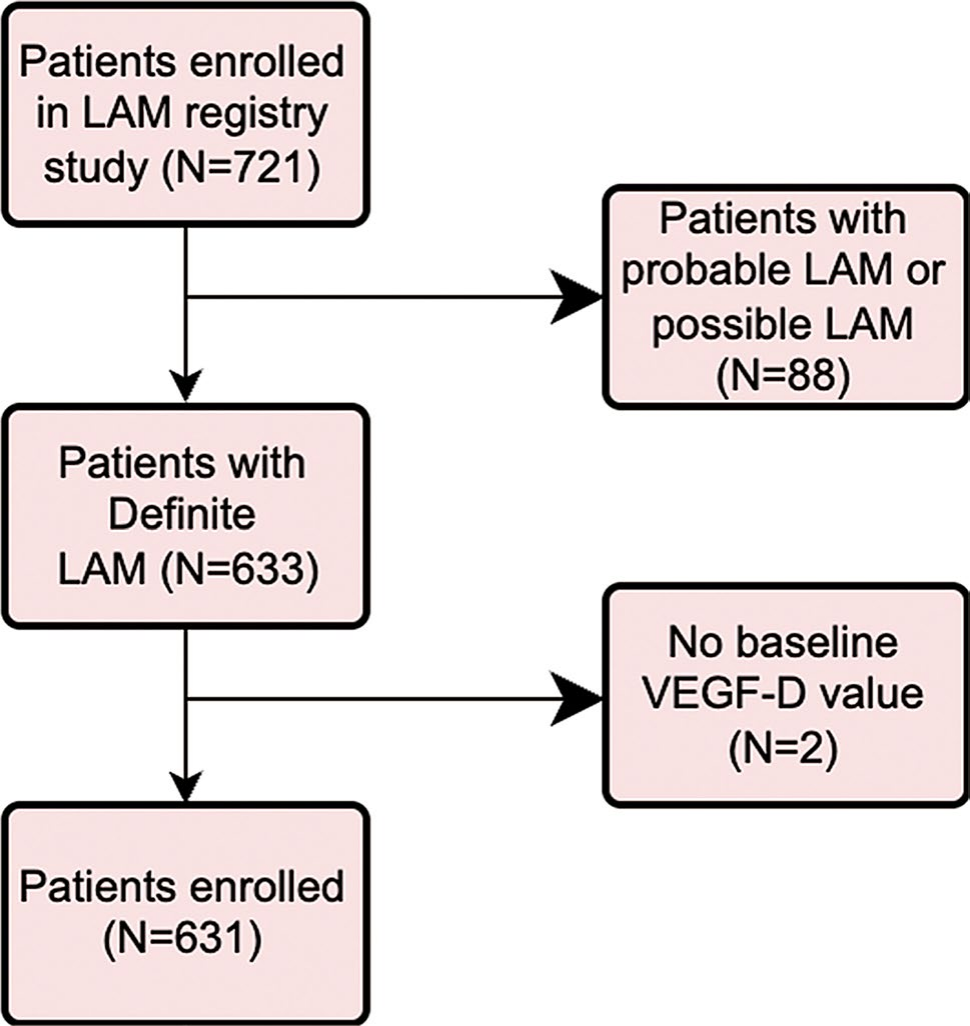
Flowchart of patient enrolment in the LAM registry study

A total of 721 patients were registered in the LAM registry study (Fig. 1). Patients with probable or possible LAM (*N* = 88) or no baseline VEGF-D value (*N* = 2) were excluded. A total of 631 LAM patients were enrolled in this study. The median level of serum VEGF-D in LAM was 1452pg/ml (820.0-2659pg/ml). Patients were categorized based on serum VEGF-D concentrations into three groups: VEGF-D non-elevated group (VEGF-D < 800pg/ml), VEGF-D mildly-elevated group (800 ≤ VEGF-D < 2000pg/ml), and VEGF-D highly-elevated group (VEGF-D ≥ 2000pg/ml). The cut-off of 800pg/ml and 2000pg/ml was selected as previously described [11, 12, 20]. There were 145 (24%), 250 (39%), and 236 (37%) individuals in each group respectively. In S-LAM, the median VEGF-D level is 1371pg/ml (794.0-2512pg/ml), whilst in TSC-LAM, the median VEGF-D level is 2315pg/ml (1190-4304pg/ml), significantly different from it in S-LAM (*P*<0.0001).

As summarized in Table 1, patients in the VEGF-D highly-elevated group tended to be younger (*P* = 0.0037) or lower BMI (*P* = 0.0158). There was no significant difference of VEGF-D levels in the time from the onset to diagnosis. The prevalence of TSC-LAM was significantly higher in the VEGF-D highly-elevated group compared to mildly-elevated group and less than 800 group (20.60% vs. 11.60% vs. 6.757%, *P* < 0.0001). The proportion of menopause was significantly lower in the VEGF-D highly-elevated group (*P* = 0.0019) whilst the proportion of smokers was higher (*P* = 0.0305). In terms of clinical manifestations, patients in the highly-elevated group exhibited a notably reduced rate of pneumothorax (*P* = 0.0278). The prevalence of angiomyolipomas (AMLs) was lower in the highly-elevated group (55.17% vs. 35.25% vs. 35.19%, *P* = 0.0004). Among 250 cases with history of AMLs, 23.64% vs. 34.25% vs. 66.22% patients had AMLs with a diameter of the largest tumor 3 cm or more in VEGF-D non-elevated group, mildly-elevated and highly-elevated group respectively. VEGF-D level was positively associated with the prevalence of AMLs with large sizes. Lymphatic involvement, including chylous effusion (5.517% vs. 14.4% vs. 25.42%) and retroperitoneal LAM (12.41% vs. 17.80% vs. 42.99%) was strongly associated with higher VEGF-D levels (*P* < 0.0001 for both). Patients with higher VEGF-D levels had significantly higher HRCT grading (33.10% vs. 60.80% vs. 59.32%, *P* < 0.0001), lower FEV_1_%pred (86.00 vs. 69.25 vs. 70.55, *P* < 0.0001), lower FEV_1_/FVC (*P* = 0.0004), lower DLco%pred (70.25 vs. 53.29 vs. 49.79, *P* < 0.0001), lower PaO_2_ (*P* < 0.0001) and higher alveoli-arterial oxygen gradient (*P* < 0.0001). The prevalence of asthma, six-minute walk distance, the SGRQ score, the need for long-time oxygen supplementation, and previous treatment of sirolimus was not different among the groups.

**Table 1:** Clinical parameters and their correlation with serum VEGF-D levels in LAM patients

	Non-elevated (VEGF-D <800pg/ml)	Mildly-elevated (800 ≤ VEGF-D <2000pg/ml)	Highly-elevated (VEGF-D ≥ 2000pg/ml)	*P*
N	145 (22.98%)	250 (39.62%)	236 (37.40%)	
Age	41.70 ± 10.91	39.95 ± 10.79	38.15 ± 8.779	0.0037^†^
Time from onset to diagnosis	15 (3–52)	18 (3–58)	21.5 (4-55.75)	0.5513^‡^
History of Tuberculosis	4 (2.759%)	13 (5.200%)	8 (3.419%)	0.9015^§^
History of Malignancy	1 (0.6897%)	4 (1.600%)	3 (1.271%)	0.6933^§^
BMI	21.85 (20.03–25.01)	21.78 (19.56-24.00)	21.00 (19.47–23.23)	0.0158^‡^
TSC-LAM	10 (6.757%)	29 (11.60%)	48 (20.60%)	< 0.0001^§^
Menopause	34 (23.45%)	44 (17.60%)	27 (11.44%)	0.0019^§^
Smoker	2 (1.379%)	10 (4.000%)	14 (5.932%)	0.0305^§^
Asthma	7 (4.828%)	19 (7.600%)	13 (5.508%)	0.9352^§^
Pneumothorax	57 (39.31%)	90 (36%)	68 (28.81%)	0.0278^§^
AMLs	80 (55.17%)	86 (35.25%)	82 (35.19%)	0.0004^§^
AMLs ≥ 3 cm	13 (23.64%)^**#**^	25 (34.25%)^**#**^	49 (66.22%)^**#**^	< 0.0001^§^
Retroperitoneal LAMs	18 (12.41%)	42(17.80%)	95 (42.99%)	< 0.0001^§^
Chylous effusion	8 (5.517%)	36 (14.4%)	60 (25.42%)	< 0.0001^§^
HRCT Grade I or II	97 (66.90%)	98 (39.20%)	96 (40.68%)	< 0.0001^§^
HRCT Grade III	48 (33.10%)	152 (60.80%)	140 (59.32%)	
FEV_1_	2.330 (1.840–2.705)	2.085 (1.523–2.528)	2.130 (1.565–2.580)	0.0239^‡^
FEV_1_%pred	86.00 (63.39–100.0)	69.25 (58.20–90.00)	70.55 (60.07–87.78)	< 0.0001^‡^
FVC	3.080 (2.810–3.402)	3.020 (2.630–3.310)	3.060 (2.685–3.415)	0.2936^‡^
FVC%pred	98.53 (88.35–108.5)	95.87 (84.45–104.2)	94.41 (86.20–106.0)	0.087^‡^
FEV_1_/FVC	71.39 (68.66–74.12)	65.41 (63.24–67.57)	64.78 (62.67–66.89)	0.0004^‡^
DLco	5.840 (4.463–6.645)	4.400 (2.985–5.843)	4.140 (2.790–5.545)	< 0.0001^‡^
DLco%pred	70.25 (55.02–80.38)	53.29 (37.99–66.89)	49.79 (37.01–63.10)	< 0.0001^‡^
PaO_2_	89.90 (85.00-96.93)	84.00 (74.98-93.00)	82.67 (72.00-91.45)	< 0.0001^‡^
P(A-a) O_2_	16.95 (8.950–23.40)	24.70 (12.30-36.25)	27.30 (14.60–40.60)	< 0.0001^‡^
6MWD	479.0 (430.0-534.8)	496.5 (450.0-540.0)	480.0 (431.0-535.0)	0.1177^‡^
SGRQ Sum	29.00 (16.25-50.00)	28.00 (13.75–42.25)	29.00 (16.00–50.00)	0.7349^‡^
Sirolimus treatment in the last year	30 (20.69%)	76 (30.40%)	40 (16.95%)	0.2193^‡^
Oxygen Therapy	4 (2.759%)	2 (0.8000%)	7 (2.966%)	0.6796^§^

TSC, tuberous sclerosis complex; retroperitoneal LAMs, retroperitoneal lymphangioleiomyomas; AMLs, angiomyolipomas; HRCT, high-resolution CT; 6MWD, six-minute walking distance; SGRQ, St. George’s Respiratory Questionnaire; †. Ordinary one-way ANOVA; ‡. Kruskal-Wallis test; §. Chi-square test for trend. Data are presented as No. (%), mean (± SD), or median (interquartile range). #. Percentage of AML size ≥ 3 cm among patients with AMLs and patients with no data of AMLs size were excluded

A more focused analysis was conducted on the subgroup of patients (*N* = 486) without prior exposure to sirolimus (sTable 1, Figure S1). Similar trends of significance were observed in parameters such as age, BMI, time from onset to diagnosis, TSC-LAM, menopause, retroperitoneal LAMs, chylous effusion, AMLs, HRCT grading, FEV_1_%pred, FEV_1_/FVC, DLco%pred, PaO_2_, and P(A-a)O_2_.

### Comparison of VEGF-D in TSC-LAM and S-LAM

The subgroup analysis of TSC-LAM and S-LAM patients within different VEGF-D levels showed distinct clinical and functional differences (sTable 2). Among the TSC-LAM subgroup, we observed significant differences in HRCT grade (*P* = 0.0049) and DLco (*P* = 0.0070) across different VEGF-D groups. Among patients with S-LAM, significant differences were observed in age (*P* = 0.0204), BMI (*P* = 0.0276), menopause (*P* = 0.0068), smoking status (*P* = 0.0049), pneumothorax (*P* = 0.0213), AMLs (*P* < 0.0001), retroperitoneal LAMs (*P* < 0.0001), chylous effusion (*P* < 0.0001), HRCT grading (*P* < 0.0001), FEV_1_%pred (*P* < 0.0001), FEV_1_/FVC (*P* < 0.0001), DLco%pred (*P* < 0.0001), PaO_2_ (*P* < 0.0001) and P(A-a)O_2_ gradient (*P* < 0.0001) and 6MWD (*P* = 0.0369) across different VEGF-D groups.

### Multiple logistic regression analysis

**Fig. 2:**
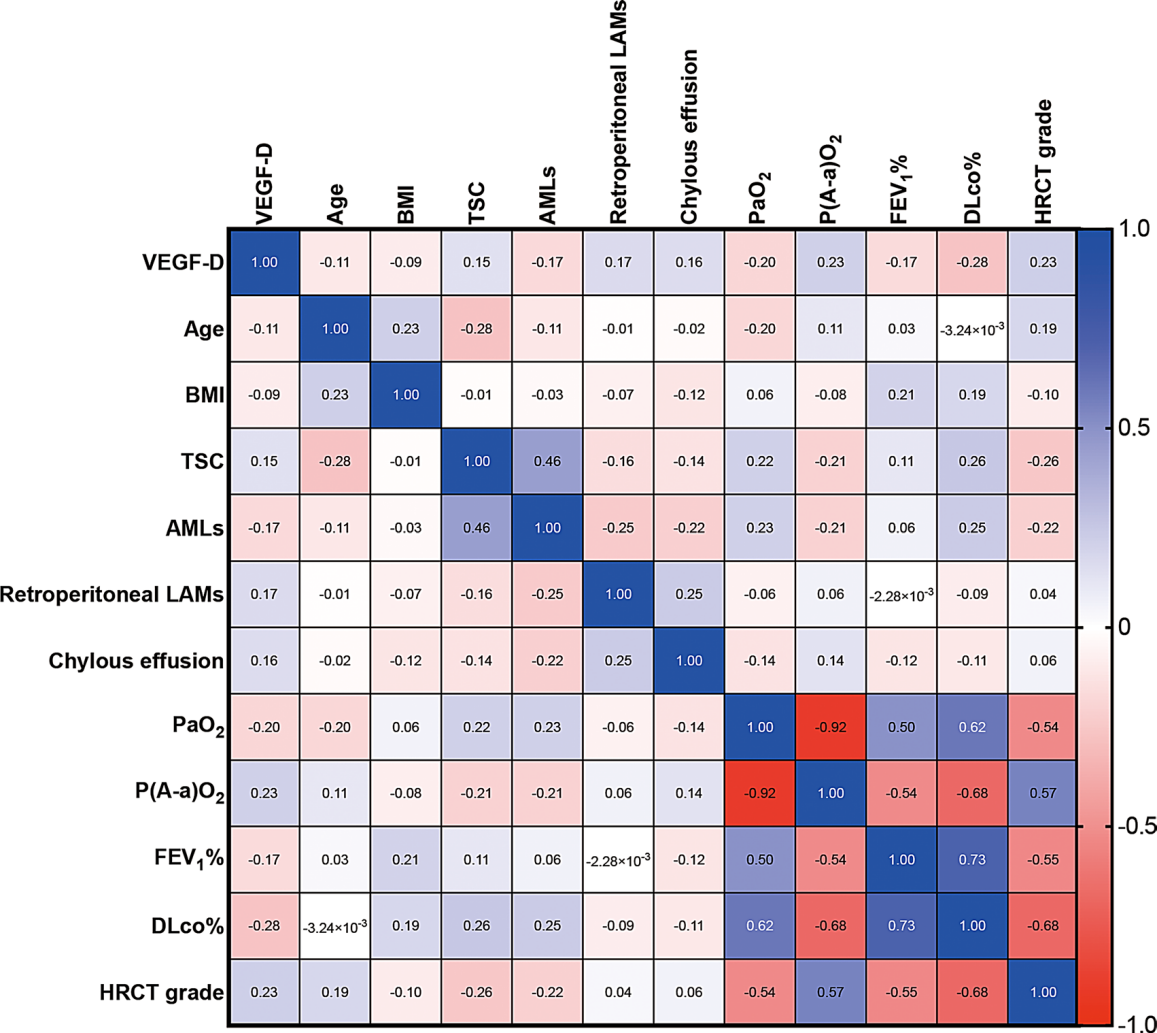
Correlation matrix heat map illustrates the relationships between VEGF-D levels and various clinical parameters in LAM patients. BMI, body mass index; TSC, tuberous sclerosis complex; retroperitoneal LAMs, retroperitoneal lymphangioleiomyomas; AMLs, angiomyolipomas; HRCT, high-resolution CT

We divided the population into 2 categories: VEGF-D level < 800pg/ml and VEGF-D level ≥ 800pg/ml. A simple logistic regression model was constructed with the VEGF-D level category as the dependent variable (Table 2). A univariate analysis showed that age, TSC, AMLs, retroperitoneal LAMs, chylous effusion, HRCT grade, FEV_1_, FEV_1_%pred, FEV_1_/FVC, DLco, DLco%pred, PaO_2_ and P(A-a)O_2_ were significant factors for VEGF-D ≥ 800pg/ml. FEV_1_, FEV_1_/FVC, DLco, DLco%pred, PaO_2_, P(A-a)O_2_ were excluded, and the remaining significant factors were included in a multiple logistic regression model. Results showed that young age, TSC, retroperitoneal LAMs, chylous effusion, and high HRCT grade were independent risk factors for VEGF-D levels ≥ 800pg/ml (Table 2). AMLs were negatively correlated to VEGF-D (Table 2). FEV_1_%pred was significantly correlated with VEGF-D in univariate analysis, but this correlation was no longer significant in multivariate analysis, potentially due to strong multicollinearity between FEV_1_%pred and the other variables in the model (Fig. 2). The model had an area under the receiver operating characteristic curve (AUC) of 0.7928, indicating good discriminatory ability.

**Table 2:** Univariate and multivariate logistic regression identifying factors associated with elevated VEGF-D levels (≥ 800pg/ml) in LAM

	Univariate logistic regression			Multivariate logistic regression		
Variable	OR	95% CI	*P*	OR	95% CI	*P*
Age	0.9752	0.9575–0.9931	0.0069	0.9741	0.9532–0.9953	0.0170
TSC-LAM	4.429	2.046–11.59	0.0006	16.07	6.744–45.04	< 0.0001
Time from onset to diagnosis	1.002	0.9992–1.005	0.1897			
History of Tuberculosis	1.592	0.5940–5.524	0.4013			
History of Malignancy	2.104	0.3701-39.50	0.4882			
Pneumothorax	0.7437	0.5078–1.095	0.1302			
AMLs	0.4371	0.2992–0.6362	< 0.0001	0.3734	0.2385–0.5829	< 0.0001
Retroperitoneal LAMs	3.089	1.861–5.405	< 0.0001	2.595	1.483–4.746	0.0012
Chylous effusion	4.215	2.119–9.628	0.0002	2.950	1.398–7.030	0.0079
HRCT Grade III	1.857	1.506–2.299	< 0.0001	2.234	1.647–3.061	< 0.0001
FEV_1_	0.617	0.4610–0.8196	0.001			
FEV_1_%pred	0.9819	0.9734–0.9903	< 0.0001	0.9985	0.9873-1.010	0.8004
FVC	0.9036	0.6454–1.261	0.5525			
FVC%pred	0.9892	0.9774–1.001	0.0732			
FEV_1_/FVC	0.9767	0.9647–0.9882	0.0001			
DLco	0.6921	0.6158–0.7742	< 0.0001			
DLco%pred	0.9671	0.9574–0.9764	< 0.0001			
PaO_2_	0.9654	0.9515–0.9788	< 0.0001			
P(A-a)O_2_	1.043	1.028–1.059	< 0.0001			
6MWD	0.9982	0.9961-1.000	0.0825			
SGRQ Sum	1.003	0.9947–1.012	0.4912			
Sirolimus treatment in past year	1.254	0.8026–2.009	0.3319			
Oxygen Therapy	0.6651	0.2131–2.484	0.5027			

TSC, tuberous sclerosis complex; retroperitoneal LAMs, retroperitoneal lymphangioleiomyomas; AMLs, angiomyolipomas; HRCT, high-resolution CT; 6MWD, six-minute walking distance; SGRQ, St. George’s Respiratory Questionnaire。

### Diagnostic pathway of LAM with VEGF-D less than 800pg/ml

**Fig. 3:**
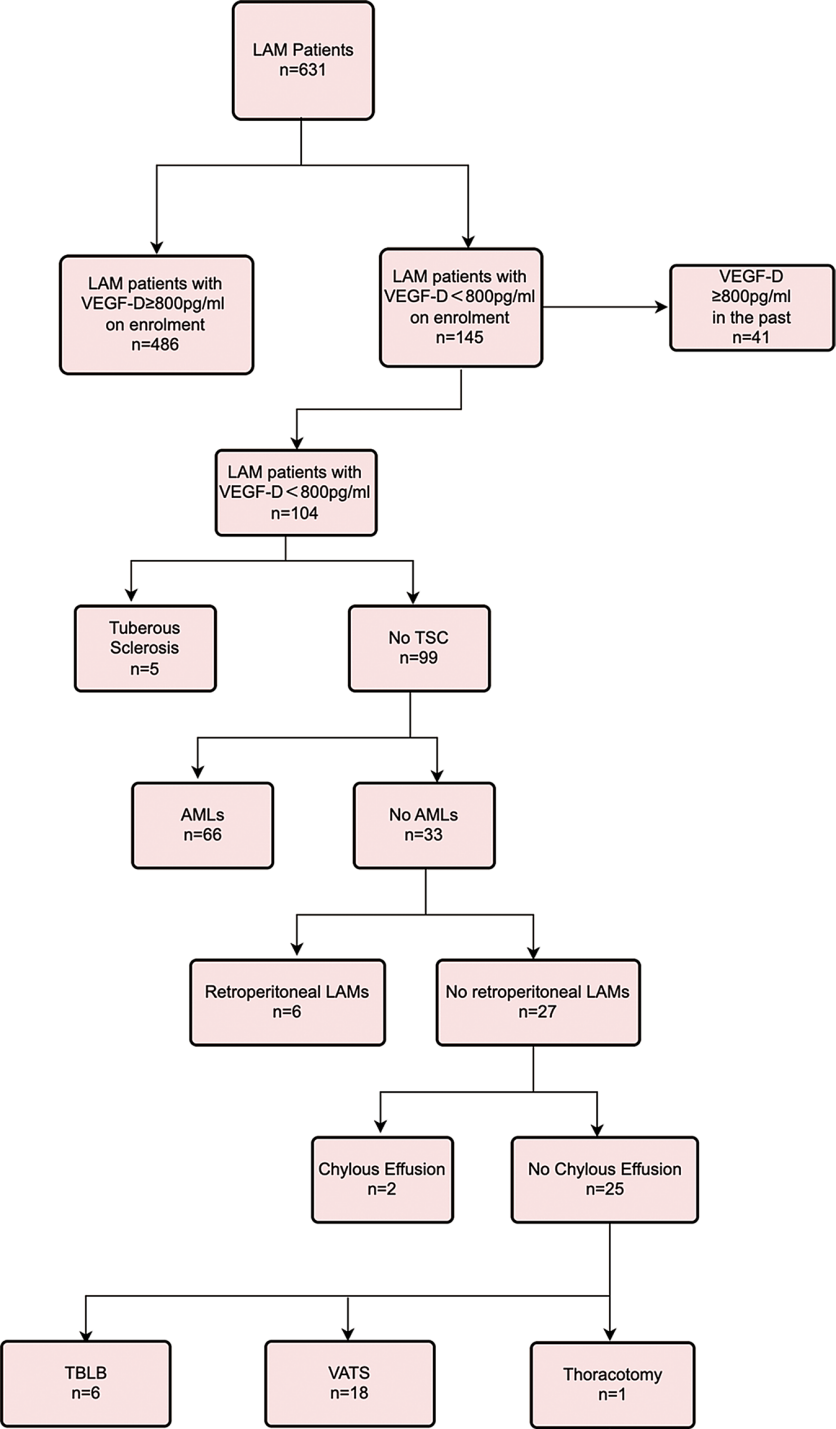
Diagnostic pathway for LAM patients with VEGF-D levels less than 800 pg/ml. DCLD, diffuse cystic lung disease; TSC, tuberous sclerosis complex; retroperitoneal LAMs, retroperitoneal lymphangioleiomyomas; AMLs, angiomyolipomas; TBLB, transbronchial lung biopsy; VATS, video-assisted thoracic surgery

The 2017 ATS/JRS guidelines for LAM include VEGF-D ≥ 800pg/ml as one of the diagnostic criteria. Therefore, majority of LAM patients can be diagnosed without lung biopsy. Interestingly, we summarized the diagnostic pathway of LAM patients in our cohort (Fig. 3). Of 631 LAM patients, 486 (77.02%) patients were diagnosed based on positive VEGF-D level on enrolment, and 41 (6.498%) patients were diagnosed based on elevated VEGF-D level before enrolment. Out of the remaining 104 patients with VEGF-D levels < 800pg/ml, 79 (75.96%) patients were able to be diagnosed through a history of TSC or coexistence of AMLs, retroperitoneal lymphangiomyomas, or chylous effusion. Only 25 (24.04% of patients with VEGF-D level < 800pg/ml, 3.96% of the total) patients achieved diagnosis based on lung biopsy (either transbronchial lung biopsy or surgery).

## Discussion

In this single-center real-world study, we investigated the clinical significance of VEGF-D. The median concentration of serum VEGF-D level was 1452pg/ml (820.0-2659pg/ml), comparable to other studies (Table 3). A total of 83.5% patients in our cohort reached VEGF-D levels 800pg/ml. Higher serum VEGF-D levels were significantly associated with lower age, lower BMI, history of smoking, premenopausal status, and higher prevalence of TSC. VEGF-D levels were associated with a lower prevalence of pneumothorax, AMLs, and a higher risk for retroperitoneal LAMs and chylous effusion, and prevalence of large size of AMLs. Functionally, higher VEGF-D levels were associated with higher HRCT grading, lower FEV_1_ and DLco showed by pulmonary function test, and lower partial pressure of oxygen. A similar trend was found in S-LAM patients but not TSC-LAM. Multiple logistic regression analysis showed that VEGF-D levels were positively associated with TSC-LAM, retroperitoneal lymphangioleiomyomas, chylous effusion, and HRCT grade, and negatively associated with age and AMLs. These findings suggest that VEGF-D may play a role in the pathophysiology of LAM and serve as a biomarker for disease severity evaluation in patients with LAM [10, 11, 21]. We also summarized the diagnostic strategy for LAM suspected patients, especially for those with VEGF-D < 800pg/ml.

**Table 3:** Summary of serum VEGF-D levels and clinical manifestations between studies

	Current study	Hirose (2019)	Amaral (2019)	Radzikowska (2015)	Xu (2013)	Young (2010)	Glasgow (2009)	Seyama (2006)
Sample size	547 S-LAM 84 TSC-LAM	92 S-LAM 16 TSC-LAM	83 S-LAM 21 TSC-LAM	36 S-LAM 12 TSC-LAM	48 S-LAM 2 TSC-LAM	56 S-LAM 28 TSC-LAM	111 S-LAM	44 S-LAM
VEGF-D **(pg/ml)**	1452 (820.0-2659)	1568 (294-10536) for S-LAM	796 (404–1588)	1807 ± 1203	3841 (2490–4000+)	1175 (780–2013) for S-LAM	1869 ± 145	1069 (809–1412)
VEGF-D in TSC-LAM (pg/ml)	2315 (1190–4304)	4485 (992–9765)	1005 (641–2732)	2682 ± 1347		3465 (1970–7195)		
Age (years)	39.68 ± 10.21	S-LAM 40 (34–47); TSC-LAM 36 (29–49)	43 ± 11	45.14 ± 10.96	40.8 ± 7.9	43 ± 12	51	37 ± 11
Pneumothorax	215 (34.07%)	S-LAM 41 (44.0%); TSC-LAM 6 (37.5%)	49 (47.1%)	24 (50%)		40 (47.62%)		
Chylous effusion	76 (12.04%)	S-LAM 5 (5.4%) TSC-LAM 0	19 (18.2%)	10 (20.83%) Chylothorax only	26 (52%)			
Renal AMLs	193 (30.59%)	S-LAM 18 (19.4%) TSC-LAM 13 (81.3%)	59 (56.7%)	21 (43.8%)	15 (30%)		40 (36%)	
Retroperitoneal LAMs	127 (20.13%)		16 (15.4%)		6 (12%)			
HRCT Grade III	245 (38.83%)			HRCT Grade 1.97 ± 0.77			38 (34%)	
FEV_1_%pred	74 (60.74–91.2)%	S-LAM 75.3 (55.1-103.9)%; TSC-LAM 80.1 (55.3–96.5)%	75 ± 26%		64.45 ± 27.33%	64 ± 23%		84.2 (73.2–95.1)%
DLco%pred	55.76 (40.45–70.10)%	S-LAM 58.8 (36.4–75.5)%; TSC-LAM 51.6 (39.5–78.3)%	74 ± 29%		48.69 ± 21.85%			
PaO_2_(mmHg)	85.27 (75.90–94.00)			79.01 ± 8.84	72.78 ± 14.73			
6MWD (m)	488.4 (440–539)		496 ± 111	522.23 ± 103.54	422.47 ± 129.72			
Sirolimus treatment	145 (22.98%)	S-LAM 33 (35.5%); TSC-LAM 2 (12.5%)	Treatment-naïve	Treatment-naïve	7 (14%)	Treatment-naïve	Treatment-naïve	Treatment-naïve
Variates associated with VEGF-D	Age, AMLs, retroperitoneal LAMs, chylous effusion, HRCT grading	Lymphangioleiomyomas, DLco baseline and decline rate	Age, DLco%	FEV_1_, DLco, PaO_2_, 6MWD, desaturation in 6MWT, HRCT grading	HRCT grading, combined chylous effusions and/or lymphatic involvement		HRCT grading, DLco, DLco/VA	DLco/VA, FEV_1_/FVC

TSC, tuberous sclerosis complex; retroperitoneal LAMs, retroperitoneal lymphangioleiomyomas; AMLs, angiomyolipomas; HRCT, high-resolution CT; 6MWD, six-minute walking distance

VEGF-D has been shown to be associated with clinical parameters and severity in LAM [10, 11, 21]. We found that patients with elevated VEGF-D levels exhibited poorer ventilation (FEV_1_, FEV_1_/FVC) and diffusion function (DLco%pred) in pulmonary function tests. The deterioration of lung function subsequently affected the partial pressure of oxygen, a finding that is similar to previous reports [22]. It was reported that VEGF-D negatively associated with FEV_1_/FVC and DLco/VA% [22, 23]. Several other studies confirmed the correlation between VEGF-D and DLco% but found no relationship between VEGF-D and FEV_1_/FVC or FEV_1_ [24, 25].

Presumably, LAM patients with more severe disease are more likely to be treated with sirolimus. We found that VEGF-D levels were not associated with sirolimus usage or not. Correlations of VEGF-D and clinical manifestations were not altered in patients treated or without treated with sirolimus. VEGF-D levels, however, will decrease after sirolimus according to our previous studies [26, 27].

Functionally, although patients with elevated VEGF-D levels seemed to have severe disease, patients did not exhibit significant deterioration in six-minute walk distance (6MWD) or St. George’s Respiratory Questionnaire (SGRQ) scores. It was reported that VEGF-D was not correlated with 6MWD [10, 25] but may be correlated with desaturation during the six-minute walk test [22]. The correlation between VEGF-D level and SGRQ scores was reported before but more validations were needed [10].

Lymphatic involvement (defined as retroperitoneal LAM or chylous effusions) was more frequent in patients with high levels of VEGF-D as previously documented [10, 12, 22, 24] suggesting the role of VEGF-D in lymphangiogenesis. The relationship between VEGF-D and AMLs is controversial (as summarized in sTable 3). Two articles reported that VEGF-D was positively associated with the size of AMLs in LAM patients [28–29]. More research studies indicate that VEGF-D is not related to or is negatively related to the occurrence of AMLs [12, 20, 22, 24, 25, 30, 31]. In this study, VEGF-D is negatively associated with AMLs in S-LAM and is not related to AMLs in TSC-LAM. However, the prevalence of AMLs equal or larger than 3 cm is positively related to VEGF-D level among patients with AMLs. Varying conclusions across different studies may attributed to several factors: (1) differences in the screening methods for AMLs; (2) differences in the composition of S-LAM and TSC-LAM cohorts enrolled; (3) potential biases due to underdiagnosis in patients with VEGF-D levels < 800pg/ml, as those with AMLs are more likely to be diagnosed, while those without AMLs are more prone to be missed. Correlationship between AML sizes and VEGF-D levels were more reasonable.

In our cohort, patients with TSC-LAM are associated with elevated levels of VEGF-D, which is highly consistent with previous research findings [20, 22, 24]. This also implies that patients with TSC-LAM may exhibit a more severe disease phenotype. A correlation between increased VEGF-D and HRCT grading as well as DLco was observed in patients with TSC-LAM. However, no relationship was found between VEGF-D and age, BMI, pneumothorax, AMLs, retroperitoneal LAMs, or chylous effusion within the TSC-LAM patients. The observed pattern may be attributed to the inherently elevated VEGF-D levels commonly found in individuals with TSC-LAM and the limited sample size of VEGF-D < 800pg/ml patients included in this study. These findings underscore the heterogeneity within the LAM population and more research was needed to validate these results.

Serum VEGF-D level is a good biomarker to discriminate LAM from other DCLD patients. Based on the ERS diagnostic criteria, the ATS/JRS guidelines introduced VEGF-D levels as a diagnostic criterion [4, 5]. We summarized the diagnostic pathway of LAM patients after the application of the ATS/JRS diagnostic pathway and only 3.96% of LAM patients had to obtain pathological evidence in the lung. The application of VEGF-D measurement in the diagnostic algorithm was able to avoid most of the lung biopsies for LAM diagnosis. As for DCLD patients with VEGF-D < 800pg/ml, systemic clinical evaluations are important for diagnosis.

The merit of the study is an analysis of data from a registry study with patient numbers over 600, and most of them were followed regularly. The limitation of the study is that we did not use VEGF-D to stratify or guide the management of LAM patients although its role in diagnosis was confirmed. Also, we use a fixed cutoff value of 800 pg/ml in diagnosis, we did not evaluate the optimal cutoff value based on the current sample size. In future, VEGF-D should be incorporated into clinical parameters to evaluate the severity of patients and optimal cutoff value for diagnosis should be further studied. The potential relationship between VEGF-D and pathological immunohistochemistry in LAM patients is an interesting area to investigate, but the current retrospective study design did not allow for direct immunohistochemical analyses of VEGF-D expression. Studies combining serum VEGF-D measurements with detailed pathological characterization would help clarify this relationship.

In summary, this study provides a detailed analysis of the relationship between serum VEGF-D levels and clinical parameters in LAM patients. Multiple logistic regression analysis showed that VEGF-D levels were positively associated with TSC-LAM, retroperitoneal lymphangioleiomyomas, chylous effusion, and HRCT grades, and negatively associated with age and AMLs. The findings underscore the importance of VEGF-D as a potential biomarker for disease severity and the need for a personalized approach to LAM management. In patients diagnosed with LAM, 83.52% was diagnosed by elevated VEGF-D levels, 12.52% was diagnosed by systemic clinical evaluation, and only 3.96% of the total patients underwent lung biopsy to achieve a definite diagnosis. The study also highlights the utility of VEGF-D levels in the diagnostic process, reducing the need for invasive procedures in a significant number of patients.

## Electronic supplementary material

Below is the link to the electronic supplementary material.

Supplementary Material 1: Online supplementary files: sFigure1, sTable 1, sTable 2 and sTable3.

## Data Availability

The data that support the findings of this study are available on request from the corresponding author. The data are not publicly available due to privacy or ethical restrictions.
