# Supplementary material for: Associations of VEGF-D levels with clinical manifestations in lymphangioleiomyomatosis: a cross-sectional analysis of 631 cases
Source: Orphanet J Rare Dis. 2025 May 26;20:255. doi: 10.1186/s13023-025-03802-4 (PMC12108032; doi:10.1186/s13023-025-03802-4)
Supplement: Supplementary file 1 — Supplementary Material 1: Online supplementary files: sFigure1, sTable 1, sTable 2 and sTable3. [file 13023_2025_3802_MOESM1_ESM.pdf]

Associations of VEGF-D Levels with Clinical Manifestations in Lymphangioleiomyomatosis: A Retrospective Analysis of 631 Cases

Luning Yang[1#], Hanghang Wang[1#], Chongsheng Cheng[1], Miaoyan Zhang[1], Danjing Hu[1], Yani Wang[1], Tengyue Zhang[1], Xiaoxin Zhang[1], Song Liu[2], Wenshuai Xu[1], Junya Liu[1], Jinrong Dai[1], Shuzhen Meng[1], Yanli Yang[1], Shao-Ting Wang[1], Xinlun Tian[1] and Kai-Feng Xu[1*]

Supplementary Figure

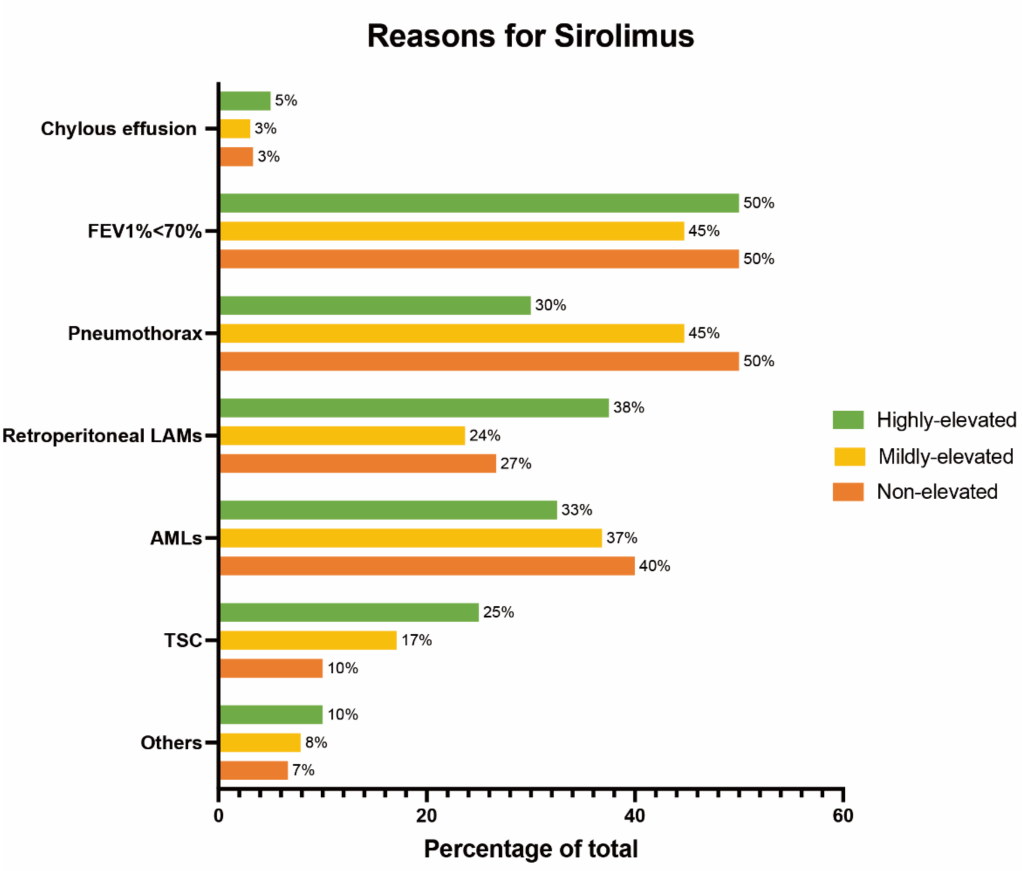

Figure S1. Reasons of sirolimus in population with different VEGF-D levels

sTable 1. Clinical parameters and correlation with VEGF-D levels in LAM patients with no exposure to sirolimus.

	Non-elevated (VEGF-D <800pg/ml)	Mildly-elevated (800≤VEGF-D <2000pg/ml)	Highly-elevated (VEGF-D ≥2000pg/ml)	*P*
**N**	116	174	196	
**Age**	41.48 ± 10.99	40.94 ± 10.95	37.67 ± 8.869	0.0006
**BMI**	22.12 (20.03-25.02)	21.68 (19.53-23.91)	20.88 (19.47-23.23)	0.0112‡
**Time from onset to diagnosis**	12.00 (2.000-42.50)	16.00 (3.000-50.75)	23.50 (4.250-60.00)	0.0350‡
**TSC-LAM**	4 (3.448%)	17 (9.770%)	43 (21.94%)	<0.0001§
**Menopause**	27 (23.28%)	32 (18.39%)	23 (11.73%)	0.0025§
**Smoker**	2 (1.724%)	8 (4.598%)	13 (6.633%)	0.1419
**Tuberculosis**	4 (3.448%)	8 (4.598%)	7 (3.571%)	0.8417
**Pneumothorax**	41 (35.34%)	58 (33.33%)	61 (31.12%)	0.7375§
**AMLs**	67 (55.76%)	54 (31.03%)	72 (36.73%)	<0.0001§
**AMLs ≥ 3cm**	11 (24.44%)	15 (33.33%)	43 (69.35%)	<0.0001§
**Retroperitoneal LAMs**	11 (9.483%)	28 (16.09%)	88 (44.90%)	<0.0001§
**Chylous effusion**	6 (5.172%)	22 (12.64%)	48 (24.49%)	<0.0001§
**HRCT Grade I or II**	89 (76.72%)	70 (40.23%)	82 (41.84%)	<0.0001§
**HRCT Grade III**	27 (23.28%)	104 (59.77%)	114 (58.16%)	
**FEV1%pred**	88.90 (69.33-101.3)	73.90 (60.65-90.03)	70.05 (61.15-87.00)	<0.0001‡
**FVC%pred**	98.26 (88.76-107.0)	95.20 (85.56-105.0)	93.85 (86.05-105.1)	0.1368‡
**FEV1/FVC**	77.85 (69.08-83.15)	69.38 (55.86-79.21)	64.78 (62.67-66.89)	<0.0001‡

**DLco%pred**	71.81 (62.55-81.40)	52.92 (39.24-67.33)	49.66 (37.29-63.67)	<0.0001‡
**PaO2**	91.00 (86.38-98.00)	83.00 (74.98-93.00)	83.05 (72.08-91.39)	<0.0001‡
**P(A-a) O2**	15.55 (8.600-20.08)	24.02 (11.93-33.25)	26.56 (15.74-39.95)	<0.0001‡
**6MWD**	501.0 (455.8-543.8)	488.0 (443.0-540.0)	474.0 (425.3-535.0)	0.0938‡
**SGRQ Sum**	23.50 (10.00-41.00)	27.00 (14.00-42.00)	27.50 (15.00-50.00)	0.2392‡

TSC, tuberous sclerosis complex; retroperitoneal LAMs, retroperitoneal lymphangioleiomyomas; AMLs, angiomyolipomas; HRCT, high-resolution CT; 6MWD, six-minute walking distance; SGRQ, St. George's Respiratory Questionnaire; †. Ordinary one-way ANOVA; ‡. Kruskal-Wallis test; §. Chi-square test for trend. Data are presented as No. (%), mean (±SD), or median (interquartile range).

sTable 2. Subgroup analysis comparing clinical parameters among TSC-LAM and S-LAM patients with varying VEGF-D levels

	TSC-LAM (n=84)				S-LAM (n=547)			
	**Non-elevated (VEGF-D <800pg/ml)**	**Mildly-elevated (800≤VEGF-D <2000pg/ml)**	**Highly-elevated (VEGF-D ≥2000pg/ml)**	***P***	**Non-elevated (VEGF-D ＜800pg/ml)**	**Mildly-elevated (800≤VEGF-D ＜2000pg/ml)**	**Highly-elevated (VEGF-D ≥2000pg/ml)**	***P***
**N**	6 (7.143%)	29 (34.52%)	49 (58.33%)		139 (25.41%)	221 (40.40%)	187 (34.19%)	
**Age**	34.17 ± 13.01	29 ± 9.352	33 ± 9.446	0.0852†	42.02 ± 10.75	41.38 ± 10.15	39.27 ± 8.287	0.0204†
**BMI**	25.50 (21.25-26.50)	21.00 (19.00-23.00)	21.00 (19.50-23.00)	0.1379‡	22.00 (20.00-25.00)	22.00 (20.00-24.00)	21.00 (19.00-23.00)	0.0276‡
**Time from onset to diagnosis**	13.00 (7.750-49.25)	120.0 (12.00-217.0)	53.00 (5.000-222.0)	0.3572‡	16 (2-53)	16 (3-48.5)	19 (4-46)	0.705‡
**Menopause**	1 (16.67%)	1 (3.448%)	4 (8.163%)	0.9615§	33 (23.74%)	43 (19.46%)	23 (12.30%)	0.0068§
**Smoker**	0 (0%)	1 (3.448%)	3 (6.383%)	0.4362§	2 (1.438%)	9 (4.072%)	11 (5.882%)	0.0449§
**Tuberculosis**	0 (0%)	2 (6.896%)	3 (6.122%)	0.7458§	4 (2.878%)	11 (4.977%)	5 (2.674%)	0.8226§
**Pneumothorax**	2 (33.33%)	7 (24.14%)	16 (32.65%)	0.647§	55 (39.57%)	83 (37.56%)	52 (27.81%)	0.0213§
**AMLs**	6 (100%)	27 (93.10%)	48 (97.96%)	0.6152§	74 (53.24%)	59 (26.70%)	36 (19.25%)	＜0.0001§

**AMLs≥3cm**	3 (60.00%)[#]	6 (27.27%)[#]	34 (77.27%)[#]	0.0040[§]	10 (20.00%)[#]	19 (37.25%)[#]	15 (50.00%)[#]	0.0047[§]
**Retroperitoneal LAMs**	0 (0%)	1 (3.448%)	6 (12.24%)	0.1279[§]	18 (12.95%)	41 (18.55%)	100 (53.48%)	＜0.0001[§]
**Chylous effusion**	1 (16.67%)	0 (0%)	2 (3.922%)	0.5914[§]	7 (5.036%)	36 (16.29%)	58 (31.02%)	＜0.0001[§]
**HRCT Grade I or II**	5 (83.33%)	24 (82.76%)	35 (71.43%)	0.0049[§]	92 (66.19%)	74 (33.48%)	61 (32.62%)	＜0.0001[§]
**HRCT Grade III**	1 (16.67%)	5 (17.24%)	14 (28.57%)		47 (33.81%)	147 (66.52%)	126 (67.38%)	
**FEV_1%pred**	73.22 (63.69-103.2)	85.80 (70.50-91.00)	81.40 (64.57-91.50)	0.6613[‡]	86.00 (63.38-100.0)	66.32 (56.83-87.45)	66.08 (57.40-87.00)	＜0.0001[‡]
**FVC%pred**	90.87 (83.18-108.3)	87.00 (80.95-102.9)	90.80 (86.12-102.8)	0.6984[‡]	99.60 (88.70-109.0)	96.20 (85.10-104.5)	94.90 (86.15-106.6)	0.1763[‡]
**FEV_1/FVC**	75.85 (68.86-79.66)	80.47 (73.23-87.08)	76.04 (70.17-83.29)	0.1375[‡]	76.81 (62.92-82.62)	65.54 (51.48-77.38)	64.49 (51.02-73.48)	＜0.0001[‡]
**DLco%pred**	61.26 (50.22-80.92)	78.68 (60.84-86.23)	63.75 (56.20-80.89)	0.1187[‡]	70.30 (55.51-80.33)	50.90 (37.05-62.78)	45.81 (32.74-58.75)	＜0.0001[‡]
**PaO_2**	91.41 (77.25-102.3)	93.00 (88.00-100.0)	92.00 (85.00-100.0)	0.8704[‡]	89.90 (85.00-96.90)	82.00 (74.00-91.00)	79.60 (70.00-88.20)	＜0.0001[‡]
**P(A-a) O_2**	15.45 (5.150-27.71)	12.00 (6.500-22.56)	15.50 (8.150-24.06)	0.7051[‡]	16.95 (9.000-23.40)	25.10 (15.05-34.95)	29.62 (19.20-41.40)	＜0.0001[‡]

**6MWD**	485.5 (285.0-521.3)	535.0 (484.5-565.0)	495.0 (450.0-552.0)	0.0698‡	497.0 (452.0-654.0)	485.0 (440.0-537.0)	470.0 (425.0-530.0)	0.0369‡
**SGRQ Sum**	14.50 (7.750-73.25)	11.00 (4.500-24.50)	19.00 (7.500-34.00)	0.3014‡	25.00 (11.00-47.00)	29.00 (18.00-45.00)	30.00 (20.00-52.00)	0.0614‡

TSC, tuberous sclerosis complex; retroperitoneal LAM, retroperitoneal lymphangileiomyomas; AMLs, angiomyolipomas; HRCT, high-resolution CT; 6MWD, six-minute walking distance; SGRQ, St. George's Respiratory Questionnaire; †. Ordinary one-way ANOVA; ‡. Kruskal-Wallis test; §. Chi-square test for trend Data are presented as No. (%), mean (±SD), or median (interquartile range). #. Pencentage of AML size≥3cm among patients with AMLs and patients with no data of AMLs size were excluded.

sTable 3. Comparison of relationship of VEGF-D levels and prevalence of angiomyolipomas among studies

Study	Sample Size	Correlation of VEGF-D and AMLs
**Glasgow (2009)**	111 S-LAM	Negatively correlated
**Dabora (2011)**	23 TSC-LAM	Positively correlated with size of AMLs
**Chang (2012)**	50 S-LAM	Unrelated
	8 TSC-LAM	
**Xu (2013)**	48 S-LAM	Unrelated
	2 TSC-LAM	
**Young (2013)**	79 S-LAM	Unrelated
	8 TSC-LAM	
**Radzikowska**	36 S-LAM	Unrelated
**(2015)**	12 TSC-LAM	
**Budde (2016)**	5 S-LAM	Positively correlated with size of AMLs
	113 TSC-LAM	
**Hirose (2019)**	92 S-LAM	Unrelated
	16 TSC-LAM	
**Amaral (2019)**	83 S-LAM	Unrelated
	21 TSC-LAM	

S-LAM, sporadic lymphangioleiomyomatosis; TSC-LAM, tuberous sclerosis complex associated with lymphangioleiomyomatosis; AMLs, angiomyolipomas.
